# Neurophysiological and Functional Assessment in Chronic Inflammatory Demyelinating Polyradiculoneuropathy (CIDP): The Correlation Between Visual Evoked Potentials and Grip Strength

**DOI:** 10.3390/reports9020096

**Published:** 2026-03-25

**Authors:** Periklis Tsoumanis, Dimitrios N. Varvarousis, Alexandra Barbouti, Theocharis Chatzoglou, Aikaterini Marini, Christos Stefanou, Panagiotis Kitsoulis

**Affiliations:** 1 Department of Ophthalmology, General Hospital of Filiates, 46300 Filiates, Greece; 2 Laboratory of Anatomy-Histology-Embryology, Medical School, University of Ioannina, 45500 Ioannina, Greece; dimvarvar@gmail.com (D.N.V.); abarbout@uoi.gr (A.B.); xatzoglou369@gmail.com (T.C.); marinikaat@yahoo.gr (A.M.); pkitsoulis@hotmail.com (P.K.); 3 2nd Breast Unit, Department of Surgery, IASO Maternity & Research Hospital, 15123 Athens, Greece; christosstefanou.gr@gmail.com

**Keywords:** CIDP, visual evoked potentials, P100 latency, grip strength, IVIg, neurophysiology, demyelination

## Abstract

**Background/Objectives**: This study investigates the relationship between Chronic Inflammatory Demyelinating Polyradiculoneuropathy (CIDP), neurophysiological markers assessed via Visual Evoked Potentials (VEPs), and functional capacity. **Methods**: A total of 190 participants, comprising 95 patients and 95 healthy controls, underwent specialized assessments of VEP latencies and amplitudes (P100 and N145), as well as dominant and non-dominant grip strength. Statistical analyses using independent-samples *t*-tests and linear regression revealed that patients exhibited significantly prolonged P100 and N145 latencies and reduced P100 amplitudes compared with controls, reflecting impaired neural conduction and heterogeneous fiber involvement. **Results**: Patients also demonstrated markedly reduced bilateral grip strength, confirming the disease’s impact on gross motor skills and sensorimotor integration. Although gender did not broadly differentiate clinical expression, patients receiving intravenous immunoglobulin (IVIg) therapy showed significant improvements in P100 latency and bilateral grip strength, compared with those not receiving treatment. **Conclusions**: These findings underscore the utility of VEPs and grip strength as reliable biomarkers for monitoring demyelination and functional status, suggesting that their combined evaluation can enhance clinical management and the assessment of therapeutic response in CIDP.

## 1. Introduction

Chronic Inflammatory Demyelinating Polyradiculoneuropathy (CIDP) is a rare, immune-mediated disorder of the peripheral nervous system, characterized by progressive or relapsing motor and sensory dysfunction. Its main pathological characteristic is immune-driven damage to myelin sheath, resulting in segmental demyelination, conduction slowing, conduction block, and, in more advanced stages, secondary axonal degeneration [1,2]. Despite advances in diagnostic standards and therapeutic options, CIDP remains a clinically heterogeneous disorder regarding disease course, severity, and response to treatment.

Traditionally, CIDP has been regarded as a disorder confined to the peripheral nervous system. However, accumulating evidence suggests that immune-mediated demyelination may not be central as well. Several recent studies reported abnormalities in central sensory pathways, including prolonged evoked potential latencies, supporting the concept of Combined Central and Peripheral Demyelination (CCPD) [3,4]. These findings challenge the classical dichotomy between central and peripheral demyelinating diseases and suggest that CIDP may involve broader neuroimmune mechanisms affecting long myelinated tracts. Such central involvement may remain clinically silent yet contribute to functional impairment and disease burden.

The diagnosis of CIDP relies primarily on clinical presentation and electrodiagnostic criteria, which focus on peripheral nerve conduction abnormalities [5]. While these criteria are essential for diagnosis, they may not fully capture the functional consequences of demyelination or the potential involvement of central pathways. CIDP is one of the commonest causes of treatable chronic neuropathy, with reported prevalence varying geographically due to differences in diagnostic criteria and case ascertainment methods [6]. Consequently, complementary tools that may detect subclinical dysfunction and quantify functional impairment are increasingly recognized as valuable adjuncts in CIDP evaluation.

Grip strength testing has emerged as a simple, reproducible, and clinically important measure of gross motor function. It reflects peripheral nerve integrity, muscle strength and overall functional capacity. It has also been correlated with disability and disease severity in neuromuscular disorders [7,8]. In parallel, Visual Evoked Potentials (VEPs) provide a sensitive and non-invasive method for assessing conduction along the visual pathways. Although CIDP primarily affects peripheral nerves, the possibility of subtle central pathway involvement provides a neurobiological rationale for using VEPs in this context. Prolongation of the P100 latency is a well-established electrophysiological marker of demyelination, while amplitude changes may reflect axonal involvement or impaired neural synchrony [9]. Because VEPs assess conduction within long central visual pathways, they may reveal subclinical alterations in neural transmission that parallel broader neurophysiological dysfunction in immune-mediated demyelinating disorders. Consequently, VEP assessment may offer additional insight into potential multilevel neural involvement in CIDP and its relationship with functional impairment.

Intravenous immunoglobulin (IVIg) therapy constitutes a first-line treatment for CIDP and has demonstrated efficacy in improving both electrophysiological parameters and functional outcomes [10,11]. However, the extent to which neurophysiological improvements translate into measurable functional recovery remains incompletely understood. In this context, the present study investigates the relationship between Visual Evoked Potential (VEP)–derived markers and grip strength in patients with CIDP, with the aim of evaluating whether integrating neurophysiological and functional assessments can improve disease characterization and therapeutic monitoring.

## 2. Materials and Methods

### 2.1. Research Design

The present study was designed to investigate the factors influencing neurophysiological function in both patients and non-patients. The sample consisted of two groups: a patient group with a clinical diagnosis of Chronic Inflammatory Demyelinating Polyradiculoneuropathy (CIDP) based on established electrodiagnostic criteria [1,2,5] and a control group comprising healthy individuals. Demographic variables, including age, sex and hand dominance, were recorded for all participants to ensure comparability between groups when evaluating functional motor performance. The control group was selected to reflect a demographic distribution similar to that of the patient group, with respect to these characteristics.

Participants underwent specialized neurophysiological assessments of Visual Evoked Potentials (VEPs), specifically measuring the latencies and amplitudes of the P100 and N145 waves. These assessment methods were conducted in accordance with the standardized protocols for evaluating visual function described by Celesia (1984) [9]. Functional motor capacity was assessed using grip strength tests of both the dominant and non-dominant hands, which serve as clinical markers of peripheral nerve integrity and motor output [7,8]. Grip strength was used as a practical functional indicator of motor performance; however, no formal adjustment was performed for additional systemic variables that may influence muscle strength, such as body mass index (BMI), fatigue status, corticosteroid use or sarcopenia. Therefore, the measurements were interpreted as overall functional indicators rather than isolated measures of CIDP-related motor impairment. To minimize measurement variability, assessments were performed using a standardized protocol and were conducted by the same examiner throughout the study, in order to reduce inter-observer variability.

Patients were diagnosed according to the diagnostic criteria of the European Academy of Neurology/Peripheral Nerve Society guidelines [10]. However, patients were not stratified by disease duration, clinical subtype (typical versus atypical CIDP), or standardized clinical severity scales, such as the Inflammatory Neuropathy Cause and Treatment (INCAT) disability score or the Medical Research Council (MRC) sum score. The present analysis primarily focused on neurophysiological and functional parameters rather than on detailed clinical stratification.

Data analysis was conducted using SPSS Statistics for Windows, Version 26.0 (IBM Corp., Armonk, NY, USA). The following statistical methodologies and criteria were employed:

- Normality Assessment: The Kolmogorov–Smirnov and Shapiro–Wilk tests were performed to determine if variables followed a normal distribution.
- Homogeneity of Variances: Levene’s test was used to ensure equal variances between groups.
- Comparative Testing: Independent-samples *t*-tests were used to assess differences between genders and the efficacy of therapeutic interventions, such as Intravenous Immune Globulin (IVIg) [10]. When the assumption of equal variances was not met, Welch’s *T*-test was utilized.
- Predictive Modeling and Reliability: Linear regression was used to estimate the relationship between age and latencies, while bootstrapping was implemented to provide stable statistical estimates.

The methodology and diagnostic approach follow the guidelines of the European Academy of Neurology/Peripheral Nerve Society [10].

### 2.2. Sample

The study sample was assembled to investigate differences between patients with the disease and individuals without the disease (controls). A total of 190 individuals participated, consisting of 95 patients and 95 controls. Participant selection was based on explicit inclusion and exclusion criteria to ensure homogeneity and the validity of the results. The inclusion criterion for the patient group was a clinical diagnosis of the disease, while for the control group, it was the absence of the disease.

Among the 95 patients with CIDP included in the study, 45 were receiving intravenous immunoglobulin (IVIg) therapy, whereas 50 were not receiving IVIg at the time of evaluation. IVIg administration followed standard therapeutic protocols: an induction dose of 2 g/kg administered over 2–5 days, followed by maintenance doses of approximately 1 g/kg every 3–4 weeks, in accordance with international treatment guidelines for CIDP [11]. However, the present analysis did not adjust for treatment duration, cumulative dosage, or baseline disease severity when comparing patients receiving IVIg with those not receiving IVIg.

Regarding demographic characteristics, the sample included participants of both genders across both groups to enable gender-based analysis. In the control group, there were 53 males and 42 females. In the patient group, 45 males and 50 females participated. These groups indicate a relatively balanced gender distribution, enhancing the comparability of the results.

The participants’ ages ranged from 30 to 70 years. In the control group, the mean age was 53.44 years (SD = 10.44), and the age distribution in the patient group was similar, allowing for intergroup comparisons without significant age-related bias. The sampling process was conducted systematically to ensure adequate representation of both genders and various age groups. This sample size and composition provide sufficient statistical power for the analysis, reducing the risk of Type II errors. However, an a priori statistical power analysis was not performed to determine the required sample size. The final sample size was determined by the number of eligible participants available during the study period.

### 2.3. Inclusion Criteria

Patient group (CIDP):

- Clinical diagnosis of Chronic Inflammatory Demyelinating Polyradiculoneuropathy (CIDP) based on established electrodiagnostic and clinical criteria
- Ability to cooperate with neurophysiological testing and grip strength assessment
- Stable clinical condition at the time of evaluation

Control group (healthy participants):

- No history or clinical evidence of peripheral or central nervous system disease
- No visual impairment that could affect Visual Evoked Potential (VEP) recordings
- Ability to undergo neurophysiological testing and grip strength measurements

General inclusion criteria applied to all participants):

- Adults aged 30–70 years
- Written informed consent provided

### 2.4. Exclusion Criteria (Applied to Both Groups)

- History of other neurological disorders (e.g., multiple sclerosis, optic neuritis, stroke, neurodegenerative diseases)
- Presence of systemic diseases known to affect peripheral nerves or visual pathways (e.g., uncontrolled diabetes mellitus, severe renal failure, connective tissue diseases)
- Known ophthalmological conditions that could affect visual pathway conduction (e.g., glaucoma, retinal disease, optic nerve pathology, or severe uncorrected refractive errors). Participants reporting visual disorders or abnormal visual acuity were excluded from the study.
- Upper limb musculoskeletal disorders limiting reliable grip strength measurement
- Current or recent (<3 months) acute infection or inflammatory condition
- Use of medications or substances known to significantly affect central or peripheral nervous system function, apart from CIDP-related treatments
- Inability to provide informed consent or comply with study procedures

### 2.5. Research Tools

Various statistical tools were employed to test the research hypotheses and elucidate relationships among variables. Initially, data distribution was evaluated using the Kolmogorov–Smirnov and Shapiro–Wilk tests to confirm normality, a prerequisite for parametric analyses. Homogeneity of variances was subsequently assessed using Levene’s test; when this assumption was violated, the independent-samples *t*-test with Welch’s correction was applied. Independent-samples *t*-tests were used as the primary inferential method to evaluate differences between groups, including comparisons between male and female patients and between patients receiving intravenous immunoglobulin (IVIg) therapy and those not receiving IVIg, with statistical significance determined using two-tailed *p*-values and supported by confidence interval estimation.

Descriptive statistical methods, including histograms, box plots, and frequency charts, were used to visualize data distributions and variability. Neurophysiological assessment was conducted using pattern-reversal Visual Evoked Potentials (VEPs), recorded in accordance with internationally accepted clinical standards, focusing on P100 latency and amplitude, and N145 latency, as established markers of demyelination and neural synchrony, based on standardized protocols described by Celesia (1984) and subsequent literature [9]. Functional motor performance was assessed via dominant and non-dominant hand grip strength using a standardized dynamometric procedure, with results expressed in kilograms, as grip strength is a validated indicator of peripheral nerve integrity and functional disability in demyelinating disorders [7,8].

Therapeutic response was evaluated by comparing neurophysiological and functional outcomes between patients receiving IVIg and those not receiving IVIg, consistent with evidence from randomized trials and international clinical guidelines demonstrating IVIg-related improvements in conduction and functional performance [10,11].

Overall, the combined use of parametric testing, assumption verification, and descriptive visualization ensured the validity and reliability of the statistical analyses and supported robust interpretation of the study findings.

### 2.6. Statistical Analysis

All statistical analyses were performed using IBM SPSS Statistics for Windows, Version 26.0 (IBM Corp., Armonk, NY, USA). The analyses were conducted between January and March 2025. Figures and graphical outputs (histograms and boxplots) were generated using the same software. Descriptive statistics are presented as means and standard deviations, and inferential analyses included independent-samples *t*-tests, Levene’s test for equality of variances, Welch’s correction where appropriate, and linear regression analyses. Linear regression was applied to explore the association between age and neurophysiological latency parameters. The analysis was conducted under the standard assumptions of linearity, independence, and normally distributed residuals, and was interpreted primarily as an exploratory assessment rather than a predictive model. Independent-samples *t*-tests were selected as the primary inferential method because the study design focused on pairwise comparisons between predefined groups (patients versus controls and IVIg-treated versus untreated patients). Although multivariate approaches such as ANCOVA or multiple regression could further adjust for potential confounding variables (e.g., age and sex), these analyses were not applied in the present study in order to maintain a straightforward analytical framework consistent with the exploratory nature of the investigation. A two-tailed *p*-value < 0.05 was considered statistically significant. In addition, exploratory two-way ANOVA analyses were performed to examine potential interaction effects between group status (control vs. CIDP) and treatment status (IVIg vs. non-IVIg). No statistically significant interaction effects were observed. No formal adjustment for multiple hypothesis testing (e.g., Bonferroni correction or False Discovery Rate control) was applied. The analyses were conducted as exploratory comparisons between predefined variables, and therefore, results should be interpreted with consideration of potential type I error inflation.

## 3. Results

**Figure 1:**
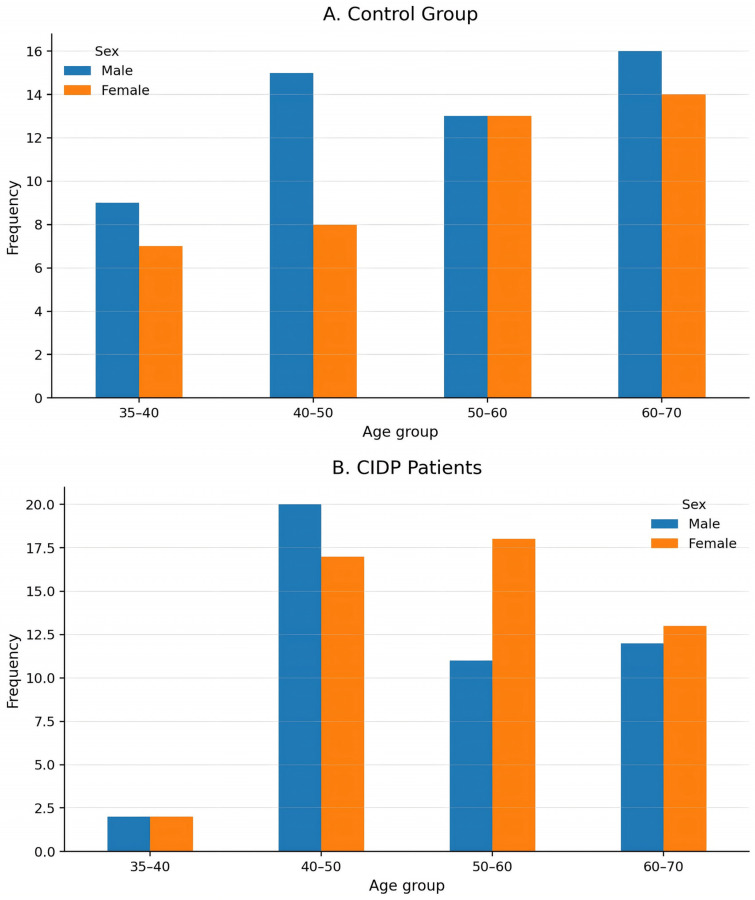
Age and sex distribution of the study population. (**A**) Control group and (**B**) patients with chronic inflammatory demyelinating polyneuropathy (CIDP).

At first the analysis focused on the demographic characteristics of the study population, specifically the age and sex distributions, to ensure comparability between the control and patient groups. As illustrated in Figure 1, participants in both groups spanned a broad age range, from approximately 30 to 70 years, providing adequate representation of middle-aged and older adults.

In the control group (Figure 1A), the mean age was 53.44 years (SD = 10.44). The age distribution appeared relatively balanced across the examined categories, with most individuals aged between 45 and 65 years. The distribution did not reveal any prominent outliers or abrupt gaps between age categories, suggesting a relatively homogeneous structure within the control population.

A comparable age range was observed in the patient group (Figure 1B). However, the distribution appeared slightly more heterogeneous, with noticeable concentrations in the 40–50 and 60–70 year age intervals. This broader dispersion may reflect the clinical variability typically observed in patients with chronic inflammatory demyelinating polyneuropathy (CIDP).

Sex distribution was also examined to further characterize the study population. In the control group, 53 participants were male, and 42 were female, indicating a relatively balanced distribution with a slight predominance of men. In contrast, the patient group showed a modest reversal of this pattern, with 50 females and 45 males, resulting in a slight female predominance. This distribution corresponds to approximately 55.8% males and 44.2% females in the control group and 47.4% males and 52.6% females in the patient group.

Overall, the two groups’ demographic characteristics show broadly comparable age ranges and relatively balanced sex distributions. These features support the sample’s suitability for subsequent statistical comparisons between controls and patients.

**Figure 2:**
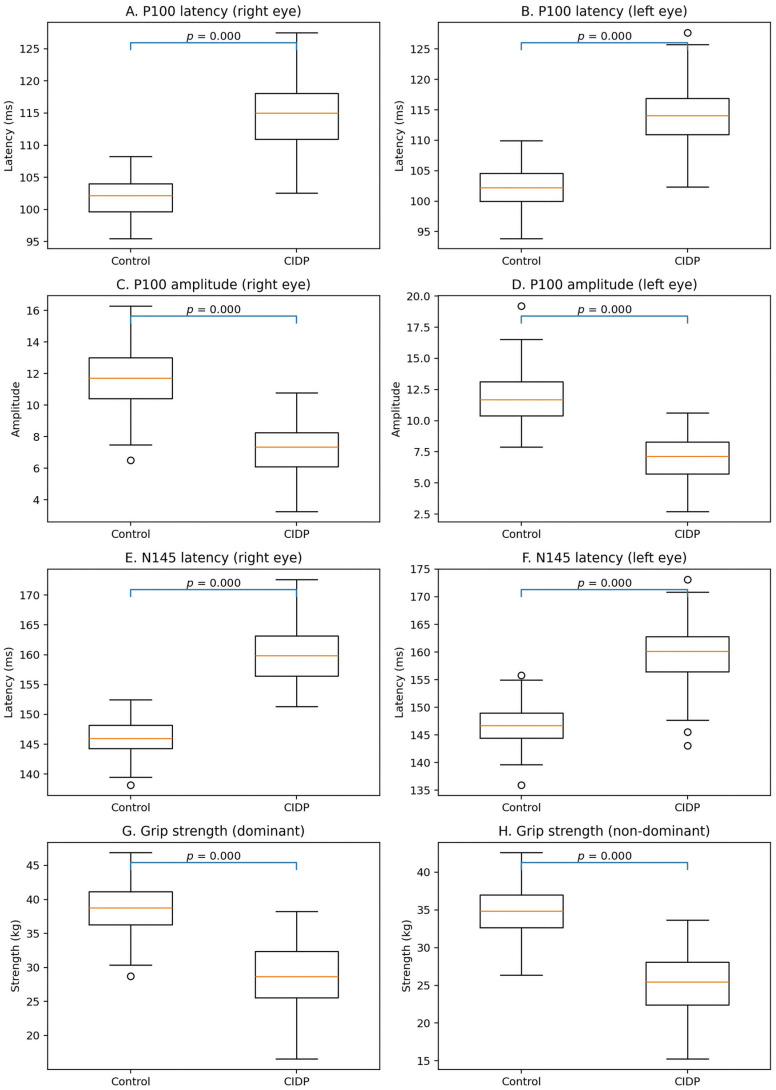
Neurophysiological and functional comparison between controls and patients with chronic inflammatory demyelinating polyneuropathy (CIDP). Panels show (**A**,**B**) P100 latency, (**C**,**D**) P100 amplitude, (**E**,**F**) N145 latency, and (**G**,**H**) grip strength for the dominant and non-dominant hands.

Following the demographic analysis, neurophysiological and functional parameters were compared between patients with CIDP and healthy controls. The results are depicted in Figure 2, illustrating the distribution of visual evoked potential (VEP) parameters and grip strength measurements across the two groups.

Patients with CIDP demonstrated a significant prolongation of P100 latency in both the right and left eyes compared with healthy controls (Figure 2A,B). In the control group, P100 latency values were concentrated around 102–103 ms, with most observations ranging between 98 and 106 ms, indicating relatively stable neural conduction. In contrast, patients exhibited clearly higher median latency values, approximately 115–116 ms, with measurements extending up to 125–128 ms, reflecting delayed conduction within the visual pathways.

In addition to latency changes, P100 amplitude was markedly reduced in patients with CIDP (Figure 2C,D). Healthy individuals showed median amplitudes of approximately 12 µV, typically ranging from 7 to 16 µV, suggesting a strong and consistent visual response. In contrast, patients demonstrated lower median amplitudes of approximately 7–8 µV, with most values ranging between 3 and 11 µV, indicating reduced neuronal synchrony and impaired functional recruitment of visual pathway fibers.

Analysis of the N145 component further supported delayed neural conduction in patients with CIDP. As shown in Figure 2E,F, the control group exhibited median N145 latency values of approximately 146–147 ms, with relatively compact distributions between 139 and 155 ms. In comparison, the patient group showed higher median latency values, near 160 ms, with latencies extending to 170–173 ms, indicating substantial slowing of visual pathway conduction, consistent with demyelinating processes.

Functional performance was evaluated through grip strength testing. Patients with CIDP exhibited significantly reduced grip strength in both the dominant and non-dominant hands compared with controls (Figure 2G,H). In the control group, the median grip strength of the dominant hand was approximately 39–40 kg, with values typically ranging from 30 to 46 kg. Conversely, patients had lower median weights of 28–29 kg, with measurements ranging from approximately 17 to 38 kg. A similar pattern was observed for the non-dominant hand, where controls showed median values of approximately 34–35 kg, compared with 25–26 kg in the patient group.

Overall, these findings indicate that CIDP is associated with both neurophysiological alterations in visual pathway conduction and measurable reductions in functional motor strength, highlighting the disease’s combined physiological and functional impact. Correlation analyses were performed using Pearson correlation coefficients to examine the relationship between VEP latency parameters and grip strength in patients with CIDP (*n* = 95). No statistically significant correlations were observed. Specifically, P100 latency in the right eye showed no significant association with dominant hand grip strength (r = 0.055, *p* = 0.599) or non-dominant hand grip strength (r = 0.028, *p* = 0.789). Similarly, P100 latency in the left eye demonstrated weak and non-significant correlations with dominant (r = −0.127, *p* = 0.219) and non-dominant hand grip strength (r = −0.122, *p* = 0.238). These findings indicate that, although group differences exist, direct linear associations between neurophysiological delay and grip strength were not statistically significant.

This difference reflects the functional impact of the demyelinating process on symmetrical muscle strength, which is not limited to the dominant hand but overall affects the performance of both upper limbs. This finding reinforces the hypothesis that the disease diffusely affects peripheral conduction and, consequently, motor function; as a result, grip strength measured in the non-dominant hand is a reliable indicator of functional severity.

Before applying parametric tests, normality of the variables was assessed using the Kolmogorov–Smirnov test with Lilliefors correction and the Shapiro–Wilk test. The results indicated that all variables were normally distributed (*p* > 0.05). Detailed results are presented in Supplementary Table S1, supporting the use of parametric statistical analyses.

Differences between male and female patients were evaluated using independent-samples *t*-tests. As shown in Supplementary Table S2, no statistically significant gender-related differences were observed in most neurophysiological or functional parameters. The only exception was N145 latency in the left eye, which demonstrated a statistically significant difference between male and female patients (*p* < 0.05). Overall, these findings suggest that the disease affects men and women in a broadly similar manner across most examined variables.

The potential effects of intravenous immunoglobulin (IVIg) therapy on neurophysiological and functional parameters were further examined in the patient group (Table 1). Among the 95 patients with CIDP included in the study, 45 were receiving IVIg therapy, whereas 50 patients were not receiving treatment at the time of evaluation. Comparisons between treated and untreated patients were performed using independent-samples *t*-tests.

Analysis of the visual evoked potential parameters revealed that patients receiving IVIg exhibited significantly shorter P100 latency in both the right and left eye compared with untreated patients (*p* = 0.026 and *p* = 0.012, respectively). These findings suggest improved neural conduction along the visual pathways in patients undergoing immunoglobulin therapy.

In contrast, no statistically significant differences were observed in P100 amplitude or N145 latency between treated and untreated patients (*p* > 0.05), indicating that these parameters were not substantially affected by treatment status within the examined sample.

Functional evaluation demonstrated that grip strength was significantly higher in patients treated with IVIg. Specifically, dominant hand strength was significantly greater in treated patients (*p* < 0.001), while a similar significant difference was also observed for the non-dominant hand (*p* = 0.015).

Overall, these findings indicate that IVIg therapy may be associated with improvements in specific neurophysiological parameters and functional motor performance in patients with CIDP, particularly in neural conduction speed and muscle strength.

## 4. Discussion

The present study sought to bridge neurophysiological markers with functional outcomes in patients with Chronic Inflammatory Demyelinating Polyradiculoneuropathy, focusing on Visual Evoked Potential (VEP) indices and bilateral grip strength measurements. Our findings demonstrate consistent, clinically meaningful differences between patients and healthy controls, supporting the concept that CIDP is associated with peripheral and potentially central conduction abnormalities that translate into measurable functional impairment.

The observed prolongation of P100 and N145 latencies is a classical electrophysiological signature of demyelination. Demyelination disrupts saltatory conduction by impairing the propagation of action potentials between nodes of Ranvier, leading to delayed signal transmission along myelinated fibers [1]. The increased dispersion of latency values observed in patients reflects heterogeneous fiber involvement, which is characteristic of immune-mediated demyelinating disorders [2]. Such temporal dispersion reduces neural firing synchrony, a phenomenon that contributes to both latency prolongation and functional inefficiency.

In addition to latency changes, the reduction in P100 amplitude observed in patients provides further insight into disease pathophysiology. Amplitude reductions are commonly interpreted as markers of impaired neural recruitment, conduction block, or secondary axonal damage [3]. The coexistence of prolonged latencies and reduced amplitudes aligns with previously described electrophysiological patterns in demyelinating diseases and is considered a sensitive indicator of combined demyelinating and axonal processes [4]. These findings highlight the importance of VEPs not only as diagnostic tools but also as biomarkers of disease severity and neural integrity.

Importantly, neurophysiological abnormalities were accompanied by significant reductions in grip strength in both dominant and non-dominant hands. Grip strength is a robust functional marker that integrates peripheral nerve conduction, muscle activation, and central motor control [5,6]. The bilateral nature of grip strength reduction highlights the diffuse impact of CIDP on motor function and supports the notion that disability in CIDP cannot be attributed solely to focal peripheral nerve involvement. On the contrary, effective motor performance requires coordinated sensorimotor integration across peripheral and central neural networks.

Although grip strength was used as a functional indicator of motor performance in the present study, other clinical disability scales or patient-reported outcome measures were not systematically applied. Consequently, the relationship between VEP abnormalities and standardized disability assessments, such as the Inflammatory Neuropathy Cause and Treatment (INCAT) disability score or other patient-reported functional outcomes, could not be evaluated. Future studies integrating electrophysiological measures, with validated clinical disability scales, may provide a more comprehensive understanding of how neurophysiological alterations may relate to patient-perceived functional impairment in CIDP.

The observed differences in VEP parameters and grip strength suggest that CIDP may exert multilevel effects along the neural axis. This observation is consistent with emerging models proposing that immune-mediated demyelination may affect long central pathways or shared antigenic targets, even in the absence of overt central nervous system symptoms [7]. Such subclinical central involvement may contribute to fatigue, reduced coordination, and impaired motor efficiency, thereby amplifying functional disability.

In addition to group comparisons, correlation analyses were performed to explore the relationship between VEP parameters and grip strength measurements. However, no statistically significant correlations were observed between electrophysiological measures and grip strength. These findings indicate that, although both neurophysiological alterations and functional impairment are present in CIDP, a direct linear association between these variables was not demonstrated in the present study.

Future studies with larger samples and more comprehensive analytical approaches may help clarify potential relationships and their clinical relevance. However, no additional imaging or electrophysiological techniques, such as brain magnetic resonance imaging (MRI) or somatosensory evoked potentials (SSEPs), were used to directly confirm central nervous system involvement. Therefore, the observed VEP abnormalities should be interpreted as indicators of altered neural conduction rather than definitive evidence of central demyelination. In addition to the group differences observed in the present study, the relationship between neurophysiological parameters and functional outcomes may also be examined through correlation analyses. Although the primary aim of the present investigation was to compare patients and controls, examining potential correlations between VEP latency measures and functional indicators, such as grip strength, could provide additional insight into the functional effects of neural conduction abnormalities. For instance, prolonged P100 latency, reflecting slowed signal transmission along visual pathways, may accompany reduced motor performance and decreased muscle strength, suggesting that conduction slowing in demyelinating conditions may affect multiple neural systems simultaneously.

**Figure 3:**
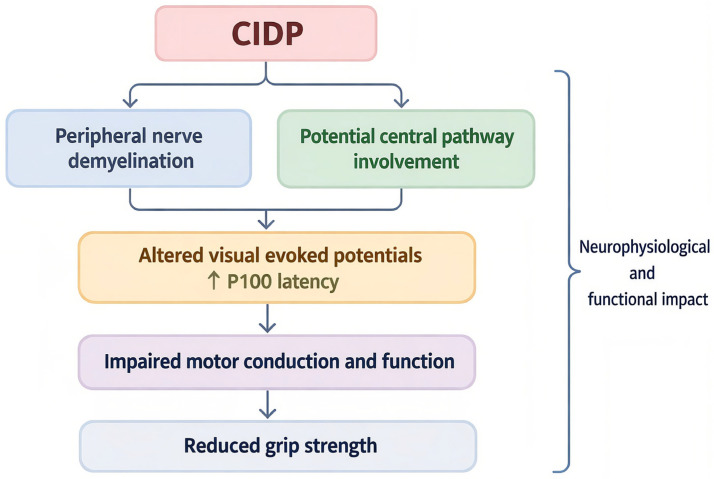
Proposed mechanistic pathway linking CIDP, delayed visual evoked potentials (P100 latency), and reduced grip strength.

From a mechanistic perspective, the relationship between altered visual evoked potentials and reduced grip strength may reflect the combined involvement of central and peripheral nervous system structures. While CIDP is primarily classified as a peripheral demyelinating neuropathy, accumulating evidence suggests that subtle alterations in central pathways may also occur in immune-mediated demyelinating diseases. In this context, delayed VEP responses may indicate impaired conduction within the central visual pathways, whereas decreased grip strength may reflect peripheral nerve dysfunction and compromised neuromuscular activation. The interaction between these mechanisms may contribute to the broader functional impairment observed in patients. A schematic representation of the proposed interaction between central visual pathway conduction abnormalities and peripheral motor dysfunction in CIDP is illustrated in Figure 3.

Analysis of demographic variables revealed no substantial gender-related differences in most neurophysiological or functional measurements, in line with prior studies reporting comparable disease expression between males and females [8]. The isolated difference observed in left-sided N145 latency may reflect biological variability or sample-specific factors rather than a systematic gender effect. Similarly, age was associated with a modest increase in P100 latency, consistent with age-related slowing of neural conduction due to physiological myelin degeneration [9]. However, the relatively weak correlation underscores that immune-mediated demyelination in CIDP outweighs age-related changes in determining neurophysiological outcomes.

Therapeutic intervention with Intravenous Immunoglobulin emerged as critical for improved outcomes. Patients receiving IVIg exhibited significantly shorter P100 latencies and greater grip strength, indicating enhanced conduction velocity and functional recovery [10,11]. These findings reinforce the established role of IVIg in modulating immune-mediated demyelination and restoring neural function. The absence of significant changes in P100 amplitude suggests that IVIg primarily improves conduction synchrony and reduces inflammatory-mediated dysfunction rather than fully reversing established axonal loss [12]. This distinction is clinically relevant, as it highlights the importance of early treatment initiation to prevent irreversible structural damage.

Collectively, these findings underscore several important implications. First, VEPs represent a sensitive tool for detecting subclinical conduction abnormalities in CIDP beyond traditional peripheral nerve studies. Second, grip strength provides a practical, meaningful functional assessment tool that reflects real-world disability. Third, IVIg therapy leads to measurable neurophysiological and functional benefits, supporting its continued role as a cornerstone of CIDP management. Finally, the integration of neurophysiological and functional assessment tools offers a comprehensive framework for disease monitoring, therapeutic evaluation, and future research.

From a clinical perspective, the present findings suggest that VEP assessment may offer adjunctive rather than stand-alone value in selected patients with CIDP, particularly when broader neurophysiological characterization or possible subclinical central pathway involvement is of interest. The observed coexistence of prolonged VEP latencies and reduced functional performance supports the notion that VEPs may provide complementary information beyond routine peripheral nerve assessment. However, these findings should be interpreted with caution, as current EAN/PNS guidelines for CIDP diagnosis and management do not establish VEPs as a standard tool for routine monitoring. Instead, VEPs may be considered a complementary method that could help refine phenotyping and support further investigation of central–peripheral interactions, rather than a procedure to be incorporated into standard follow-up practice at the present stage of evidence.

Future studies integrating neurophysiological measurements, functional performance assessments, and advanced neuroimaging approaches may further elucidate the pathways linking visual system abnormalities with motor dysfunction in CIDP. In addition, schematic representations of these potential central–peripheral interactions may facilitate a clearer understanding of how immune-mediated demyelination affects neural conduction across multiple levels of the nervous system.

By adopting a multilevel assessment strategy, this study contributes to a more nuanced understanding of CIDP as a disorder with systemic rather than exclusively peripheral consequences. Such an approach provides improved clinical monitoring, facilitates individualized treatment strategies, and guides future investigations for the mechanisms underlying central–peripheral interactions in immune-mediated demyelinating diseases.

This study has several limitations that should be considered when interpreting the findings. First, the observational and cross-sectional design precludes causal inference; while associations between neurophysiological parameters, grip strength, and treatment status (including IVIg therapy) were identified, longitudinal changes and definitive treatment effects cannot be conclusively established. Second, although the sample size was adequate for statistical analysis, the patient group was clinically heterogeneous with respect to disease duration, severity, and treatment exposure, which may have contributed to variability in neurophysiological and functional measures. Third, recruitment was conducted at a single center, which may limit the generalizability of the results to broader or more diverse populations.

Additionally, although participants with known ophthalmological disorders were excluded, detailed ophthalmological examinations or retinal imaging were not systematically performed. Therefore, the possibility that subtle or subclinical ocular factors could influence VEP measurements cannot be completely excluded. Similarly, additional neurophysiological or imaging techniques capable of directly assessing central nervous system involvement, such as brain magnetic resonance imaging (MRI) or somatosensory evoked potentials (SSEPs), were not incorporated in the present study. Consequently, while the observed VEP abnormalities may suggest altered neural conduction, they cannot be interpreted as definitive evidence of central demyelination.

Finally, while Visual Evoked Potentials and grip strength provide robust and clinically relevant markers, they do not capture all aspects of functional disability or the complex interactions between central and peripheral neural systems in CIDP. Future multicenter, longitudinal studies incorporating additional functional, neurophysiological, and imaging biomarkers are warranted to confirm and extend these findings.

## 5. Conclusions

In conclusion, the investigation confirms that Visual Evoked Potentials (VEPs) and grip strength are reliable, non-invasive indicators of neurophysiological and functional status in patients with Chronic Inflammatory Demyelinating Polyradiculoneuropathy (CIDP). Our study demonstrates that while gender does not significantly alter the clinical or physiological presentation of the disease, therapeutic intervention with Intravenous Immunoglobulin (IVIg) provides measurable improvements in both neural conduction times and bilateral motor power.

The strong relationship between delayed sensory pathway responses and reduced muscle strength underscores the diffuse impact of CIDP across the entire nervous system. These findings suggest that a multilevel assessment approach—integrating electrophysiological markers with functional motor tests—provides a more comprehensive framework for early detection, clinical monitoring, and the objective assessment of therapeutic responses.

## Figures and Tables

**Table 1:** Effect of Intravenous Immunoglobulin (IVIg) Treatment on Neurophysiological and Functional Outcomes.

Variable	Equality of Variances	F	Sig.	T	df	Sig. (2-Tailed)	Mean Diff.	SE	95% CI Lower	95% CI Upper
P100_latency_R_disease	Assumed	0.3	0.585	2.26	93	0.026	2.28	1.01	0.28	4.28
	Not assumed			2.26	89.34	0.026	2.28	1.01	0.27	4.29
P100_latency_L_disease	Assumed	0.07	0.790	2.56	93	0.012	2.57	1.00	0.57	4.56
	Not assumed			2.56	89.70	0.012	2.57	1.00	0.57	4.56
P100_amp_R_disease	Assumed	0.02	0.896	−0.87	93	0.387	−0.29	0.34	−0.96	0.38
	Not assumed			−0.87	90.35	0.387	−0.29	0.33	−0.96	0.37
P100_amp_L_disease	Assumed	2.30	0.133	1.46	93	0.149	0.48	0.33	−0.18	1.14
	Not assumed			1.43	82.71	0.156	0.48	0.34	−0.19	1.15
N145_latency_R_disease	Assumed	0.28	0.596	−0.65	93	0.519	−0.64	0.99	−2.60	1.32
	Not assumed			−0.65	88.24	0.521	−0.64	0.99	−2.61	1.33
N145_latency_L_disease	Assumed	0.55	0.460	0.00	93	0.998	0.00	1.12	−2.22	2.21
	Not assumed			0.00	87.72	0.998	0.00	1.12	−2.23	2.23
Grip_dom_disease	Assumed	0.04	0.848	−4.04	93	<0.001	−3.69	0.91	−5.50	−1.88
	Not assumed			−4.02	87.79	<0.001	−3.69	0.92	−5.51	−1.87
Grip_non_dom_disease	Assumed	0.17	0.681	−2.47	93	0.015	−2.06	0.84	−3.72	−0.40
	Not assumed			−2.48	91.34	0.015	−2.06	0.83	−3.71	−0.41

## Data Availability

The original contributions presented in this study are included in the article and Supplementary Material. Further inquiries can be directed to the corresponding author.

## Supplementary Materials

The following supporting information can be downloaded at: https://www.mdpi.com/article/10.3390/reports9020096/s1, Table S1: Tests of Normality for Neurophysiological and Functional Variables.; Table S2: Gender-Based Comparison of Neurophysiological and Functional Parameters in the Patient Group.

## Abbreviations

CIDP	Chronic Inflammatory Demyelinating Polyradiculoneuropathy
VEP	Visual Evoked Potential
VEPs	Visual Evoked Potentials
P100	Positive wave occurring approximately 100 milliseconds after stimulus onset
N145	Negative wave occurring approximately 145 milliseconds after stimulus onset
IVIg	Intravenous Immunoglobulin
IVIG	Intravenous Immunoglobulin (alternative capitalization used in some contexts; standardized to IVIg in this manuscript)
CNS	Central Nervous System
CCPD	Combined Central and Peripheral Demyelination
SD	Standard Deviation
df	Degrees of Freedom
kg	Kilograms
ms	Milliseconds
µV	Microvolts
SPSS	Statistical Package for the Social Sciences
IBM	International Business Machines Corporation
