# Supplementary material for: Neurophysiological and Functional Assessment in Chronic Inflammatory Demyelinating Polyradiculoneuropathy (CIDP): The Correlation Between Visual Evoked Potentials and Grip Strength
Source: Reports (MDPI). 2026 Mar 25;9(2):96. doi: 10.3390/reports9020096 (PMC13108045; doi:10.3390/reports9020096)
Supplement: Supplementary file 1 [file reports-09-00096-s001.zip › reports-4173685-supplementary.pdf]

Table S1. Tests of Normality for Neurophysiological and Functional Variables.

Variable	Kolmogorov-Smirnov[a] Statistic	Kolmogorov-Smirnov[a] df	Kolmogorov-Smirnov[a] Sig.	Shapiro-Wilk Statistic	Shapiro-Wilk df	Shapiro-Wilk Sig.
**P100_latency_R_Non_disease**	**0.067**	**95**	**0.200***	**0.982**	**95**	**0.229**
**P100_latency_R_disease**	**0.050**	**95**	**0.200***	**0.991**	**95**	**0.777**
**P100_latency_L_Non_disease**	**0.038**	**95**	**0.200***	**0.993**	**95**	**0.916**
**P100_latency_L_disease**	**0.078**	**95**	**0.194***	**0.991**	**95**	**0.761**
**P100_amp_R_Non_disease**	**0.064**	**95**	**0.200***	**0.993**	**95**	**0.908**
**P100_amp_R_disease**	**0.070**	**95**	**0.200***	**0.988**	**95**	**0.540**
**P100_amp_L_Non_disease**	**0.055**	**95**	**0.200***	**0.977**	**95**	**0.099**
**P100_amp_L_disease**	**0.058**	**95**	**0.200***	**0.990**	**95**	**0.664**
**N145_latency_R_Non_disease**	**0.045**	**95**	**0.200***	**0.990**	**95**	**0.730**
**N145_latency_R_disease**	**0.066**	**95**	**0.200***	**0.979**	**95**	**0.127**
**N145_latency_L_Non_disease**	**0.060**	**95**	**0.200***	**0.987**	**95**	**0.448**

N145_latency_L_disease	0.062	95	0.200*	0.988	95	0.529
Grip_dom_Non_disease	0.046	95	0.200*	0.991	95	0.790
Grip_dom_disease	0.070	95	0.200*	0.988	95	0.520
Grip_non_dom_Non_disease	0.058	95	0.200*	0.990	95	0.718
Grip_non_dom_disease	0.043	95	0.200*	0.989	95	0.598

a.: Lilliefors Significance Correction *: This is a lower bound of the true significance.

Table S2. Gender-Based Comparison of Neurophysiological and Functional Parameters in the Patient Group.

Variable	Equality of Variances	F	Sig.	t	df	Sig. (2-tailed)	Mean Difference	Std. Error Difference	95% Conf. Interval Lower	95% Conf. Interval Upper
P100_latency_R_disease	Equal variances assumed	0.02	0.881	-0.148	93	0.882	-0.153	1.03	-2.204	1.897
	Equal variances not assumed			-0.148	91.53	0.883	-0.153	1.03	-2.206	1.900
P100_latency_L_disease	Equal variances assumed	2.28	0.134	-0.646	93	0.52	-0.667	1.03	-2.718	1.383
	Equal variances not assumed			-0.639	84.70	0.525	-0.667	1.04	-2.744	1.409
P100_amp_R_disease	Equal variances assumed	4.08	0.046	1.306	93	0.195	0.434	0.33	-0.226	1.095
	Equal variances			1.322	91.60	0.189	0.434	0.32	-0.218	1.087

	**not assumed**									
**P100_amp_L_disease**	**Equal variances assumed**	**0.07**	**0.780**	**0.969**	**93**	**0.335**	**0.321**	**0.33**	**-0.337**	**0.981**
	**Equal variances not assumed**			**0.967**	**91.14**	**0.336**	**0.321**	**0.33**	**-0.339**	**0.982**
**N145_latency_R_disease**	**Equal variances assumed**	**2.84**	**0.095**	**0.929**	**93**	**0.356**	**0.911**	**0.98**	**-1.038**	**2.862**
	**Equal variances not assumed**			**0.940**	**91.69**	**0.35**	**0.911**	**0.97**	**-1.015**	**2.839**
**N145_latency_L_disease**	**Equal variances assumed**	**4.40**	**0.038**	**2.080**	**93**	**0.04**	**2.263**	**1.08**	**0.102**	**4.4250**
	**Equal variances not assumed**			**2.117**	**88.42**	**0.037**	**2.263**	**1.06**	**0.138**	**4.389**
**Grip_dom_disease**	**Equal variances assumed**	**0.42**	**0.515**	**0.961**	**93**	**0.339**	**0.944**	**0.98**	**-1.006**	**2.894**
	**Equal variances not assumed**			**0.963**	**92.42**	**0.338**	**0.944**	**0.98**	**-1.003**	**2.892**
**Grip_non_dom_disease**	**Equal variances assumed**	**0.02**	**0.895**	**1.230**	**93**	**0.222**	**1.048**	**0.85**	**-0.644**	**2.740**
	**Equal variances not assumed**			**1.233**	**92.75**	**0.221**	**1.048**	**0.84**	**-0.639**	**2.736**
